# Multi-Solvent Extraction Procedure for the Pioneer Fecal Metabolomic Analysis—Identification of Potential Biomarkers in Stable Kidney Transplant Patients

**DOI:** 10.3390/diagnostics11060962

**Published:** 2021-05-26

**Authors:** Soumaya Kouidhi, Nessrine Souai, Muhanad Alhujaily, Oumaima Zidi, Ameni Kochbati, Alaeddine Redissi, Tareg M. Belali, Imene El Kossai, Jamelddine El Manaa, Ameur Cherif, Wissem Mnif, Amor Mosbah

**Affiliations:** 1 LBVBGR_LR11ES31, High Institute of Biotechnology of Sidi Thabet, University of Manouba, Ariana 2020, Tunisia; nessrine.souai@fst.utm.tn (N.S.); oumaima.zidi@hotmail.fr (O.Z.); amenikoch22@gmail.com (A.K.); redissialadin@gmail.com (A.R.); ameur.cherif@uma.tn (A.C.); amor.mosbah@gmail.com (A.M.); 2 Department of Clinical Laboratory, College of Applied Medicine, University of Bisha, P.O. Box 551, Bisha 61922, Saudi Arabia; malhujaily@ub.edu.sa; 3 Faculty of Applied Medical Sciences, University of Bisha, 255, Al Nakhil, Bisha 67714, Saudi Arabia; blaly@ub.edu.sa; 4 Unit of Organ Transplant, Military Training Hospital, Tunis 1008, Tunisia; imenelkossai@gmail.com (I.E.K.); jamel_manaa@yahoo.fr (J.E.M.); 5 Department of Chemistry, Faculty of Sciences and Arts in Balgarn, University of Bisha, P.O. Box 199, Bisha 61922, Saudi Arabia; 6 Laboratory of Biotechnology and Valorisation of Bio-GeoRessources, Higher Institute of Biotechnology of Sidi Thabet, BiotechPole of Sidi Thabet, University of Manouba, Ariana 2020, Tunisia

**Keywords:** metabolomics, kidney transplantation, metabolites, biomarkers

## Abstract

Metabolic alteration plays a functional role in kidney allograft complications. Metabolomics is a promising high-throughput approach in nephrology but is still limited by the lack of overlap in metabolite coverage. We performed an untargeted fecal metabolomic analysis of forty stable kidney allograft recipients and twenty non-transplant controls. First, we applied the ultra-high performance liquid chromatography (UHPLC) analysis coupled with the Diod Array detector. The potential biomarkers were then collected and identified by gas chromatography-mass spectrometry (GCMS). In order to allow for complete coverage of the fecal polar and non-polar metabolites, the performance of five organic solvents with increasing polarity was investigated successively. UHPLC analysis revealed that the fecal metabolite profiles following the five extractions were significantly different between controls and kidney allografts. GC-MS analysis showed that the best predictors’ metabolites belonged mainly to long-chain fatty acids, phenolic compounds, and amino acids. Collectively, our results showed the efficiency of our pioneer method to successfully discriminate stable kidney-transplant recipients from controls. These findings suggest that distinct metabolic profiles mainly affect fatty acid biosynthesis and amino acid metabolism. In such a context, the novel insights into metabolomic investigation may be a valuable tool that could provide useful new relevant biomarkers for preventing kidney transplant complications.

## 1. Introduction

It is well known that renal transplantation and monitoring success closely depend on controlled immunosuppression [1]. Because of long-term immunosuppressive therapies, transplant patients could face several risks: nephrotoxicity, diabetes, hyperlipidemia, hypertension, and atherosclerosis [2]. Nevertheless, relatively simplistic clinical measurements remain in use for the monitoring of renal transplant patients. Thus, to monitor patients’ outcomes and to improve the efficacy and safety of immunosuppressive therapy, innovative approaches able to reflect the interindividual pharmacodynamic variability responses are needed.

Recently, the emergence of unbiased metabolomic profiling allowed a deeper comprehension of an organism’s physiological state. Therefore, many metabolomic approaches aim to maximize metabolite coverage to identify candidate biomarkers, reflecting dynamic phenotype under a specific set of environmental conditions [3,4]. Several studies focused on exploring fecal metabolic profiling, as it is a noninvasive and information-rich sample type [5,6]. Reported data gave new insights about host-gut co-metabolites and therefore host-gut microbiota interactions [7]. To date, however, only a few metabolomic studies have been applied to studying the modification of the intestinal milieu and the deficit of gut-metabolite excretion under renal impairment [8]. Additionally, the fecal metabolic profile of kidney disease patients has rarely been explored.

Furthermore, it remains challenging to simultaneously extract all metabolite classes using a single method [9,10]. Although significant efforts have been devoted to metabolomic extraction, there is not a universal solvent recipe for a highly efficient extraction because of the differences in samples and analysis methods. Therefore, it is necessary to develop a mixture of extraction solvents for the simultaneous analysis of polar metabolites and lipids in feces. Biphasic solvents, which separate metabolites into polar and non-polar fractions, are widely used [11].

Relevant studies have focused on the increased link between gut microbiota and kidney diseases (the gut-kidney axis) [12,13]. The gut microbiota is now recognized as a highly metabolically active community of microorganisms and a critical regulator of its host homeostasis. However, disruption of the normal gut microbiota may lead to dysbiosis, which could dictate the pathophysiological phenotype of chronic kidney disease (CKD) [14].

Thus, in this study, we enrolled kidney graft recipients undergoing immunosuppressive therapy and nontransplant controls to investigate the fecal metabolic profile in this population using untargeted ultra-performance liquid chromatography (UPLC). Several research studies reported a metabolomic comparison between kidney graft recipients and healthy controls to assess the shift in certain metabolic pathways induced by renal transplantation using serum or urine samples [15]. However, little is known about whether fecal metabolites can predict kidney allograft status. The identification of significantly different metabolites was performed using gas chromatography–mass spectrometry (GCMS) analysis.

Our purpose was to develop a sample extraction method that maximizes both polar and non-polar metabolites, and to identify specific biomarkers. To this end, the goal of this work was to perform, for the first time, a successive five-solvent-based metabolite extraction protocol. Interestingly, this method allowed for the identification of the ideal solvent for setting up potential specific biomarkers.

## 2. Materials and Methods

### 2.1. Volunteer Recruitment and Sample Collection

Subjects were enrolled from the Military Hospital for a fecal specimen collection and data study. A total of forty patients with stable kidney allograft (KT group) (12 females, 28 males, mean age 42 years), and twenty healthy control subjects (control (T) group) (10 females, 10 males, mean age 44 years) were enrolled in this study. The subjects provided the fecal specimens within one day of production and the samples were frozen at −80 °C. Clinical and demographic data of study groups are further detailed in Supplementary Table S1.

All subjects gave their informed consent for inclusion before they participated in the study. The study was conducted in accordance with the Declaration of Helsinki, and the protocol was approved by the Ethics Committee of the Military Hospital of Tunis (N °05032018).

### 2.2. Fecal Metabolite Extraction

This protocol describes the simultaneous extraction of polar and non-polar metabolites from fecal samples. Respecting a decreasing gradient of solvent polarity, we performed a successive five-solvent-based (Ethanol-Ethyl, Acetate-Diethyl, Ether-Chloroform-Hexane) metabolite extraction protocol. All fecal samples were aseptically taken. Before analysis, fecal water was extracted via mixing 3 g of fresh stool with Ethanol (stored at −20 °C) in the ratio 3:20 (g:mL; feces:Ethanol). The mixtures were homogenized for 3 min and centrifuged at 4000 rpm for 20 min at 4 °C. The supernatants were transferred and filtered through a 0.45 µm Millex-GV Syringe Filter. An amount of 20 mL of Ethyl Acetate was added to the pellets. The mixtures were well shaken, vortexed for 3 min and centrifuged at 4000 rpm for 20 min at 4 °C. The same operation was repeated successively when adding Diethyl ether, Chloroform, and Hexane. A triplicate extraction was performed for every sample. All filtered fecal waters were dried to complete dryness of solvents, under reduced pressure in a speed vacuum at 10 °C, to get a pellet of concentrated metabolites. All the extracted metabolites were stored at −20 °C until analysis.

### 2.3. Sample Preparation

A total of 2 mg of dried metabolites were reconstituted in the same solvent of extraction and homogenized until the sample was completely dissolved, then adjusted to 1 mL with the same solvent. The mix was subsequently filtered through syringe filters (0.45 μm pore size).

### 2.4. UHPLC Analysis

The metabolites were detected with a UHPLC Thermo UltiMate 3000 system, coupled with a Diod Array detector from 190 nm to 1100 nm (DAD RS 3000 and RS 3400 MWD) (Thermo Scientific™; Dionex™ UltiMate™ 3000; US) and fluorescence detection (FLD 3100 and RS 3400 FLD HR) (Ultimate 3000) using a C18 column ((4.6 × 250 mm 5 µm), C/N5020- 03946, S/N 3FF37044 GL Sciences Inc. Tokyo Japan) in a column compartment (TCC 3000 SD and RS TCC 3000). The UHPLC was equipped with an auto injector (WPS 3000 WPS 3000 SL and RS), a pump (SRD 3 × 00) opted for a flow rate of 1 mL/min, and the temperature set at 30 °C with an injection volume of 10 μL. Wavelengths were set as an indicator at 210, 280, 350, and 450 nm. A total of 10 μL of the sample was eluted with a mobile phase composed of HPLC water/Trifluoroacetic acid (0.08%) and Acetonitrile (Sigma-Aldrich) with a (95/5%) gradient following these steps—Step 1: 5% Acetonitrile for 2 min; step 2: From 5% Acetonitrile to 90% Acetonitrile in 20 min; step 3: 95% Acetonitrile for 2 min; and finally, step 4: 5% Acetonitrile for 2 min. The QC strategy procedure adopted in this paper is based on the use of blank, calibration, and control samples. Blank and calibration samples allowed for the control of the performance of the HPLC instrument (Thermo Scientific™; Thermo UltiMate 3000 system, US), while control samples were included in the analytical batch and treated in the same way as the samples [16].

### 2.5. Peak Identification by GC/MS

UHPLC has been demonstrated as a powerful, robust, sensitive, and selective method for the simultaneous quantification of various compounds. For a good separation with UHPLC, some controllable factors, including flow rate, eluent additives, pH, analyte nature, type of mobile phase, type of stationary phase, the content of sample matrices, type and settings of the detector, and temperature must be enhanced. With the aim of a good UHPLC separation, these factors mentioned above were optimized for the analysis of our samples. We performed several runs of the same sample (10 times) and we collected the same peak several times. A co-elution of the different collected peaks was performed to ensure that the same peak had been collected.

After reaching the equilibrium, the sample was injected into the sample loop and 21 global UHPLC (Thermo Scientific™; Thermo UltiMate 3000 system, US) chromatograms were chosen. Each sample was subjected to two independent HPLC (Thermo Scientific™; Thermo UltiMate 3000 system, US) runs to validate the metabolomic profile and to have the right retention time for the 21 reproducible peaks that were significantly different between the two groups and could be suggested as potential metabolic biomarkers. Fractions were manually collected throughout the running UHPLC chromatogram. Every identified peak was collected separately, and a triplicate was performed for every peak. All the fractions were dried under reduced pressure in a speed vacuum at 45 °C. To further identify the 21 metabolites, the dried fractions were analyzed using the Agilent GC 7890B-MS 240 (Agilent, CA, USA) ion trap gas chromatography (GC) technology equipped with an MS detector. The GC-MS analysis was performed in two steps:

For the first step, each sample pellet was diluted with 500 µL of the extraction solvent. The mixtures were filtered through a 0.22 µm Millex-GV Syringe Filter (Millex^®^ Syringe Filters; Merck KGaA, Darmstadt, Germany). All the samples were run on GCMS with a 500 µL blank of each extraction solvent.

The second step was the derivatization: Each sample was re-dried and derivatized by adding 800 µL of N-Hexane and 400 µL of (1 M) Sodium methylate to the metabolite pellets. The resulting solution was then vortexed, 200 µL of H_2_SO_4_ (0.1 M) was added, and the mixture was homogenized. After decantation, 500 µL of the supernatants were transferred to GC-MS glass vials. A blank with MilliQ water was prepared and treated the same as the derivatized samples.

### 2.6. GC-MS Analysis

The samples were analyzed using the Agilent GC 7890B–MS 240 (Agilent, CA, United States) ion trap Gas Chromatography technology equipped with MS detector (GC-MS). Injections were in a splitless mode for 0.75 min, using a 2 mm I.D. non-deactivated direct liner. The separation was carried out on an HP-5MS capillary column (30 m × 0.250 mm; 0.25-μm film thickness). The analysis was carried out in full scan mode for 60 min. Autosampler injected 1 µL of each sample and the separation was performed using the column in split mode and with the ionization range from 50 to 1000 mV. The carrier gas was helium with a flow rate of 1.1 mL/min. The injector temperature was set at 280 °C and GC oven temperature was programmed at 40 °C for 2 min, then a slope from 50 °C up to 250 °C maintained for 20 min. The analysis was carried out in full scan mode for 60 min.

### 2.7. Identification and Comparison of Volatile Compounds

Mass spectral data processing and metabolite identification were performed using Automated Mass Spectral Deconvolution and Identification System (AMDIS) (AMDIS-version 2.71, 2012) and the National Institute of Standards and Technology (NIST) (version 2.0, 2011) database. The detected metabolite peaks were identified using three components within NIST; these were a match of >800, a 90% probability of a match to NIST library standards and a head-to-tail comparison of the fragments. A compound was considered to be present when it satisfied these 3 criteria. This process provided relative ion abundance; therefore, no units of ion abundance are available. A compound with a similarity index of more than 80% was considered as a potential biomarker [17], therefore compounds that were found in less than 20% of the entire sample cohort were removed from further analysis.

### 2.8. Statistical Analysis Approaches for Metabolic Change Detection

The UHPLC data sets multivariate statistical analysis was conducted using SIMCA-P version 12.0 software package (Umetrics, Umeå, Sweden). First, the unsupervised principal component analysis (PCA) was performed to observe intrinsic clusters and find obvious outliers. Then, the supervised orthogonal projection to least squares discriminant analysis (OPLS-DA) was employed to visually discriminate between KT patients and healthy controls. The OPLS-DA model removes variability not relevant to class separation. Thus, only one predictive component is normally used for the discrimination between two classes. OPLS-DA was used to differentiate metabolite profiles between different extraction methods. For the model validation parameter Q2 (the fraction of variations of the X and Y matrices explained by the model; the X matrix was the metabolite features, and the Y matrix was the treatment groups), values above 0.4 were indicative of a robust model, i.e., true differences between the comparing groups, and Q2 between 0.7 and 1.0 indicated that the model was highly robust. R2X (R2Y) indicated the fraction in which the X (Y) matrix was explained by the model. The statistical model was tested for robustness with the use of a CV-ANOVA (analysis of variance in the cross-validated residuals of a Y variable) from SIMCA. The difference between the KT and CT groups was calculated with unpaired sample *t*-test (Student’s *t*-test) (Excel 2019). All features with *p*-values below 0.05 indicated that these features can indeed be regarded as potential “biomarkers”.

## 3. Results

### 3.1. Patient Characteristics

To assess the effects of changes in the metabolic profile of the gut microbiota, we performed an untargeted metabolomic analysis of fecal contents using UHPLC protocol. Fecal metabolite profiling was analyzed in 40 kidney graft recipients vs. healthy subjects. The demographic and clinical characteristics of the kidney graft recipients are summarized in Table 1 and supplementary Table S1.

### 3.2. Metabolomics Workflow

**Figure 1:**
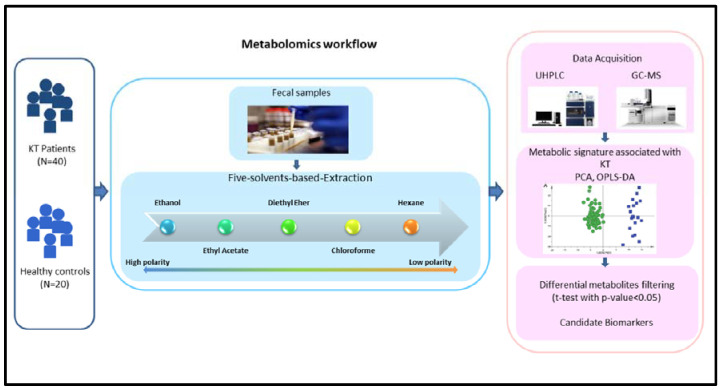
Workflow of UHPLC-GC/MS-based metabolomics for metabolomic profiling and data interpretation of fecal samples from stable kidney transplant patients.

**Figure 2:**
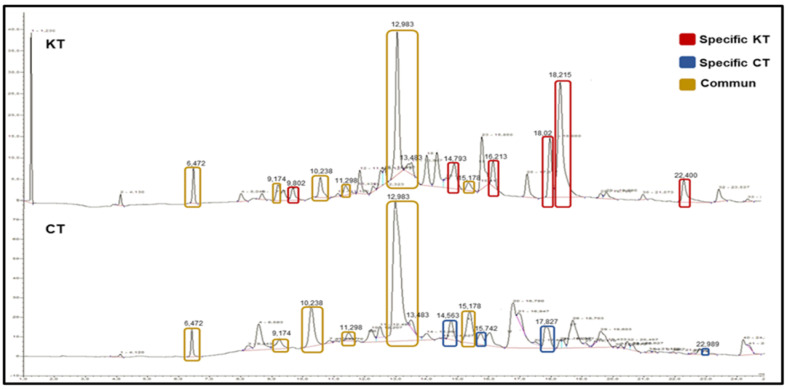
Representative fecal UHPLC spectra with retention time of samples from kidney transplant patients (KT) and healthy volunteers (CT). UHPLC analysis with C18 column, 30 °C, UV Diod Array detector from 190 nm to 1100 nm. The mobile phase used was HPLC water/Trifluoroacetic acid (0.08%) and Acetonitrile with a 95:5 gradient, flow rate of 1 mL/min. Yellow rectangles highlight common RPs between KT and CT groups. Red rectangles highlight specific RPs of the KT group. Blue rectangles highlight specific RPs of the control (T) group.

**Figure 3:**
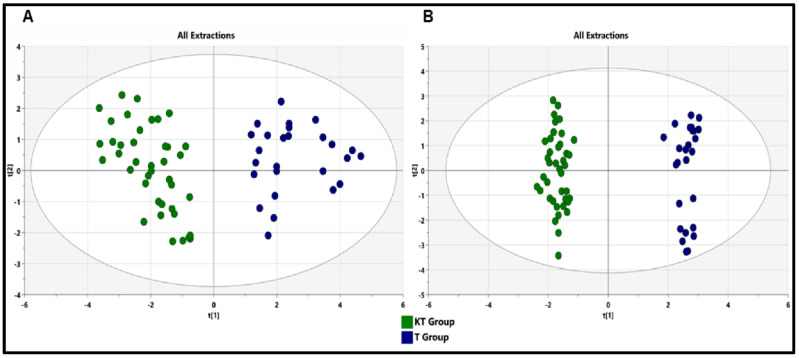
PCA and OPLS-DA score plots of the fecal metabolic profiles from the KT and control (T) groups. An overview of the data from all the five extractions confirms that there are no outlying samples within a 95% confidence interval. (**A**) PCA−score plot model, with values of R2 = 0.504 and Q2 = 0.315. Blue circles represent healthy control samples and green circles represent KT samples (**B**) Orthogonal partial least squares discriminant analysis (OPLS-DA)—score plot model showing separation based on all extraction methods, with R2(X) = 0.347, R2(Y) = 0.983, Q2 = 0.904, and cross-validated analysis of variance (CV−ANOVA) *p* = 2.31 × 10^−23^ values. Blue circles represent healthy control samples and green circles represent KT samples.

**Figure 4:**
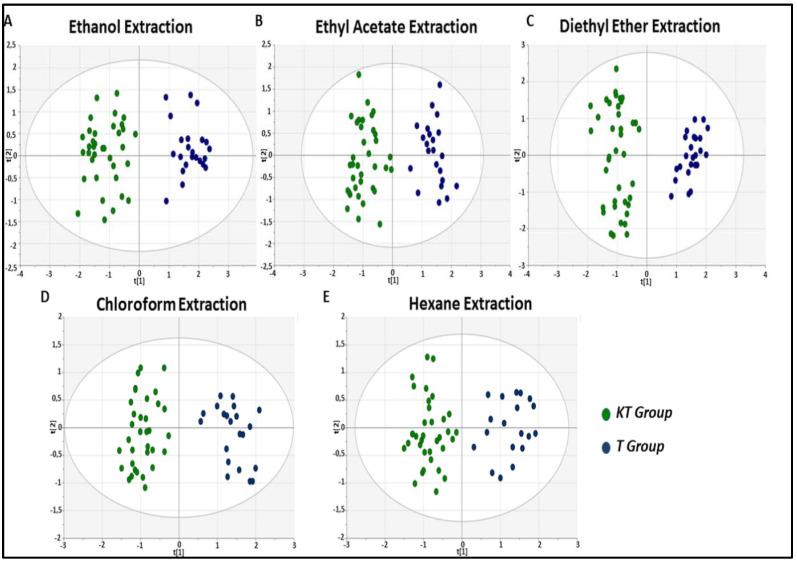
OPLS-DA score plots of the fecal metabolic profiles from the KT and control (T) groups. An overview of the data from each extraction confirms that there are no outlying samples within a 95% confidence interval. Green circles represent KT samples and blue circles represent healthy control samples. (**A**) OPLS-DA score plot model showing discrimination of stable KT patients from controls based on the Ethanol extraction method, with R2(X) = 0.602, R2(Y) = 0. 881, Q2 = 0.71, and CV-ANOVA *p* = 2.85 × 10^−9^ values. (**B**) OPLS-DA score plot model showing discrimination of stable KT patients from controls based on the Ethyl Acetate extraction method, with R2(X) = 0.291, R2(Y) = 0.871, Q2 = 0.718, and CV-ANOVA *p* = 1.56 × 10^−14^ values. (**C**) OPLS-DA score plot model showing discrimination of stable KT patients from controls based on the Diethyl ether extraction method, with R2(X) = 0.486, R2(Y) = 0.938, Q2 = 0.815, and CV-ANOVA *p* = 2.02 × 10^−24^ values. (**D**) OPLS-DA score plot model showing discrimination of stable KT patients from controls based on the Chloroform extraction method, with R2(X) = 0.505, R2(Y) = 0.889, Q2 = 0.791, and CV-ANOVA *p* = 1.07 × 10^−12^ values. (**E**) OPLS-DA score plot model showing discrimination of stable KT patients from controls based on the Hexane extraction method, with R2(X) = 0.341, R2(Y) = 0.873, Q2 = 0.798, and CV-ANOVA *p* = 9.63 × 10^−19^ values.

**Figure 5:**
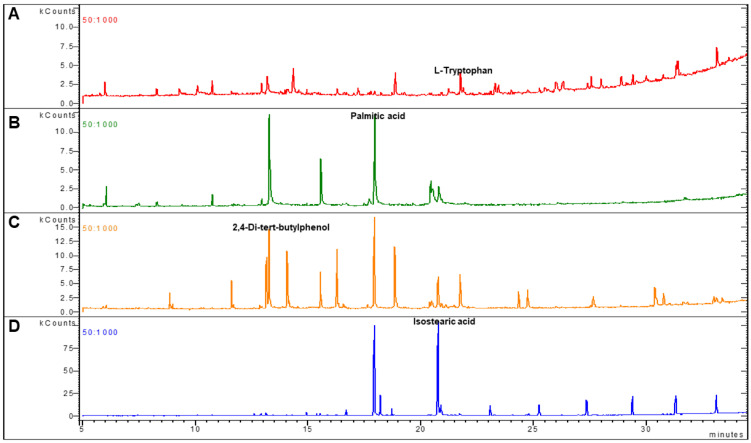
GC/MS chromatograms (TIC) of the best predictor metabolites: l-Tryptophan (**A**), Palmitic acid (**B**), Butylphenol (**C**), and Isostearic acid (**D**). Agilent GC 7890B–MS240 ion trap gas chromatography (GC) technology equipped with MS detector. Metabolites were isolated through an HP-5MS capillary 54 column (30 m × 0.250 mm i.d.; 0.25-μm film thickness; Agilent J&W Scientific).

Figure 1 shows the schematic workflow of our global metabolomics study. Fecal samples were collected from 40 KT patients and 20 Healthy controls. Metabolites were extracted from the fecal samples following the gradient of solvent polarity and analyzed using the UHPLC platform; the metabolite identification was subsequently performed using GC-MS approach. To discriminate KT patients from healthy subjects, all metabolic features were analyzed in the principal component analysis (PCA) and orthogonal partial least square discrimination analysis (OPLS-DA) (Figure 2, Figure 3, Figure 4 and Figure 5). The potential biomarkers were further extracted using the criteria of VIP > 1 and FDR ≤ 0.05.

### 3.3. Dynamic Changes in Unknown Fecal Metabolic Patterns in Kidney Transplant Patients

To analyze changes in the metabolic pattern, which are associated with the kidney transplantation process and therapy, we performed a successive extraction of both polar and non-polar metabolites with five solvents to ensure a full coverage of metabolites, and thereby running separate phases after each extraction on the UHPLC. We used two typical analytical procedure steps (as often used in metabolomics). Following the successive five-solvent extraction from the feces, a total of approximately 95 different reproducible peaks (RPs) or features of whole polar and non-polar metabolites were detected using untargeted UHPLC (Figure 2).

The metabolomic signature showed dramatic changes in response to immunosuppressive therapy. The unsupervised PCA was initially utilized on the identified peaks and the scatter plots using the score of the first principal component (PC1) and the second principal component (PC2) for each sample. As we can see, the PCA model showed a clear trend of group clustering between the kidney transplant group and the control healthy group (Figure 3A). To maximize the group separation and identify discriminating metabolites, the supervised OPLS-DA classification model, using one PLS component and one orthogonal component, was established. The OPLS-DA method was able to correctly separate the whole fecal metabolome of healthy subjects from kidney transplant patients (Figure 3B). Furthermore, the CV-ANOVA test was performed to examine the statistical significance of the differences between the two groups in the OPLS-DA model, which resulted in a score of *p* = 2.31 × 10^−23^, indicating that the differences between the groups within the model were highly significant. Goodness of fit values and predictive ability values (R2X, R2Y, and Q2) were 0.347, 0.983, and 0.904, respectively. These values indicated that the model possessed a satisfactory fit with good predictive power. Globally, both PCA and OPLS-DA analyses revealed that the two groups had unique metabolome profiles. Furthermore, to assess differences in the metabolic structure among patients undergoing kidney graft over time, we divided our cohort into the following subgroups according to the post-graft period: short post-graft period (“SG” from 3 months to 1 year; n = 11), medium-length post-graft period (“MG” from 1 year to 10 years; n = 20), and long post-transplant period (“LG” from 10 to 22 years; n = 9). The OPLS-DA plot reported a total discrimination between the CT group and the different graft periods (SG, MG, and LG), but no significant separation according to the post-graft period (Supplementary Figure S1).

To better delineate the fecal metabolic alterations between healthy and kidney allograft subjects, we separately analyzed the five extractions. For each extraction, the unsupervised PCA analysis clearly showed a significant separation between the two studied groups, and the established OPLS-DA model exhibited a good ability to discriminate from each other as well (Figure 4). Interestingly, the number of detectable reproducible peaks (RPs) decreased according to the decrease of the polarity of the solvent. Ethanol extraction showed the highest detectable RPs (n = 36 RPs). Additionally, we reported 32 detectable RPs for the Ethyl acetate extraction, 28 detectable RPs for the Diethyl ether extraction, and 26 detectable RPs for the Chloroform extraction, Hexane extraction showed the lowest detectable RPs (n= 23 RPs).

### 3.4. Fecal Metabolic Differences between Kidney Transplant Patients and Healthy Individuals and the Selection of Potential Biomarkers

To assess the potential utility of altered metabolites as predictive markers of potential biomarkers of kidney transplant patients undergoing immunosuppression therapy, relevant RPs were selected between the control and kidney transplant groups using the Student’s *t*-test. When a *p*-value is below 0.05, the variable is considered to contribute to the difference between the two groups. First, we investigated the total dataset from all the extractions. As shown in Table 2, a total of 21 differential RPs in feces were picked as potential biomarkers of kidney transplant patients.

To gain further insights, the significantly different 21 UHPLC fractions were collected separately, and then analyzed using a GC/MS approach that was shown to achieve a comprehensive metabolic fingerprint with good analytical characteristics (Figure 5). After excluding the solvents’ metabolites, the 21 metabolites were identified and listed in Table 2.

As shown in Table 2, the 21 metabolites could mainly be classified into 9 fatty acids and long-chain fatty acids, 3 phenolic compounds, 2 amino acids, and 7 other classified metabolites. The identified metabolites mainly correspond to the alterations of biosynthesis of unsaturated fatty acids and tryptophan metabolism.

Subsequently, the most relevant metabolites were summarized considering the five extractions separately, as mentioned in Table 2. Interestingly, the ethanol extraction, followed by the Ethyl acetate and the diethyl ether extractions, allowed for the highest number of differential metabolites that could be suggested as potential biomarkers separating the two groups. Furthermore, ethanol extraction covered the biggest range of metabolite polarity.

## 4. Discussion

Currently, metabolomics is a promising tool for the study of the metabolic profile in renal disease, allowing for the potential identification of relevant biomarkers in kidney transplantation management and therapy [18,19]. To the best of our knowledge, this is the first untargeted metabolomic analysis investigating fecal metabolome in renal function decline, especially in stable KT patients receiving immunosuppression therapy.

Recent data have focused on the serum and urine metabolic signature of kidney transplant patients [20]. Several mechanisms, such as uremic toxins or an alteration in enzyme activity, have been suggested to link blood or urine metabolites to an impaired renal function [21,22]. Fecal samples have recently been thought of as a good choice for the study of metabolism since they can be collected easily and noninvasively [23]. Likewise, metabolomic analyses of feces could increase our understanding of the mechanisms underlying gut microbiome–host interactions in the kidney transplantation state [24]. However, to date, metabolomic analyses of fecal samples in these patients remain elusive.

Our purpose was to develop an untargeted metabolomic extraction method for the analysis of fecal metabolic fingerprint that could enable us to discriminate enrolled KT patients from controls. Until now, only a few studies have dealt with the optimization of the method used for the sample preparation of feces. To maximize metabolite coverage, we optimized, for the first time, a five-solvent-based method to extract both polar and non-polar metabolites simultaneously from the same samples. This could be extremely beneficial as it avoids much of the variation that can occur when extracting both types of metabolites separately from different samples. Taken together, our results showed that fecal metabolic profiles of KT patients issued from all the five extractions were different from the control group. The same results were found when the five extractions were analyzed separately. These findings are in line with our recently published study investigating metabolic profiles of kidney transplant recipients using the GC-MS analysis approach [25]. The present results indicate that our concurrent analysis approach could successfully distinguish between groups through the statistical analysis of the profiling data. Overall, our findings are in line with previous reports which demonstrated that both urine and plasma metabolomic signatures can discriminate between grafts and healthy subjects [26]. Furthermore, according to our GC-MS analysis, the identified metabolites vary profoundly in polarity. They range from hydrophilic, polar metabolites with low molecular weight, to hydrophobic, non-polar high-molecular-weight metabolites. This diversity means that our method is an efficient extraction approach that could potentially be used for the clinical recovery of the whole fecal metabolome and for biomarker discovery. Interestingly, our findings showed the efficiency of the pioneer extraction of fecal metabolites using at least three different solvents with increasing polarity: highly polar, intermediate, and non-polar. Ethanol, diethyl ether, and Ethyl acetate extractions allowed for the highest number of differential metabolites. As such, most of the recent studies have tried to improve metabolite extraction and profiling, focusing on either the polar metabolites or on lipids. However, few studies, if any, have performed protocols that are effective at simultaneously extracting both polar and non-polar metabolites [27,28]. Moreover, there is still little information in the literature and lack of a universal approach to sample treatment for fecal metabolic profiling [29].

Using VIP and FDR values from OPLS-DA, a total 21 RPs were selected as the best predictors from the different extractions. The 21 RPs indicating significant differences in metabolic profiles between the kidney transplants and controls could be potential clinical biomarkers. Thus, the corresponding metabolites of the selected 21 RPs were then deeply analyzed by GC-MS. Among these identified metabolites, we reported long-chain fatty acids, phenolic compounds, and amino acids. Nevertheless, these relevant metabolites that clearly distinguish stable transplant recipients from controls may correspond mainly to alterations of the biosynthesis of unsaturated fatty acids, tryptophan metabolism, or gut microbial metabolism. These results are in line with previous observations and are likely to yield new insights into kidney transplant outcomes.

Based on the metabolite selection step, fatty acids and long-chain fatty acids, including dodecanoic acid, valeric acid, palmitic acid, octadecanoic acid, isostearic acid, erucic acid, and oleic acid, were significantly altered in KT. At this point, altered fatty acids may be a marker of the progressing organism wasting in the course of kidney failure. This is consistent with most earlier studies that have focused on the lipid nephrotoxicity hypothesis based on Moorhead’s work [30]. There are pieces of evidence that fatty acid dysregulation can contribute to the alteration of renal function [31]. Indeed, several reports have shown that excess fatty acids, such as palmitic acid and stearic acid, accompanied by triglyceride accumulation can damage the renal tissue that facilitates the progression of nephropathy [32], especially when associated with obesity and diabetes. Lipotoxicity induced by saturated FAs (SFA), including palmitic and stearic acids, causes insulin resistance and cell death. Furthermore, a recent report has demonstrated the marked elevation of plasma-free fatty acids and saturated fatty acids in the pre-hemodialysis blood samples from end-stage renal disease patients as compared to controls [33]. An interesting in vivo study showed a significant impact of altered fatty acid metabolism in advanced chronic kidney disease [34].

Interesting studies also reported that the tryptophan pathway is involved in chronic kidney diseases. Recently, it has been demonstrated that tryptophan depletion together with the accumulation of tryptophan-related toxic metabolites are associated with kidney function decline and disease progression [35]. Moreover, emerging evidence has shown that kidney function could be indicated by the ratio between plasma kynurenic acid and tryptophan [36]. In this clinical context, several enzymes have been proposed to influence the tryptophan pathway, such as the enzymes kynurenine aminotransferase (KAT), indoleamine 2,3-dioxygenase (IDO) in the kidney and/or tryptophan 2,3-dioxygenase (TDO) in the liver [37]. Serum and urinary levels of tryptophan and kynurenic acid have recently been used as a prognostic and for monitoring the renal transplant function [38]. In a previous UPLC/MS-based metabolic profiling in patients undergoing hemodialysis, a total of 19 differential fecal metabolites were identified and correspond mainly to alterations of tryptophan metabolism, lysine degradation, and beta-alanine metabolism [39]. Recent study-based urine metabolomics revealed that 14 differential metabolites identified distinguished acute rejection from stable transplant recipients and showed high sensitivity and specificity for the diagnosis of renal allograft recipients with acute rejection [40]. In another recent report, Bassi et al. showed a correlation between the glomerular filtration rate (GFR) and the serum concentration of tryptophan, glutamine, and dimethylarginine isomers. They found the same association between GFR and urinary levels of histidine, DOPA, dopamine, carnosine, SDMA, and ADMA [41]. Furthermore, urinary and blood levels of tryptophan and kynurenic acid have been suggested as important parameters in the prognosis of renal transplant function [35,38].

Nonetheless, no previous reports have assessed the fecal metabolite profiles of stable renal allografts, although dysbiosis in gut microbiota was previously reported to be involved in the progression of various kidney diseases [42,43]. Dysbiosis is often observed in uremic states, especially characterized by the retention of TMAO [44] and uremic toxins (p-cresyl sulphate and sulphate), which derive from the imbalanced metabolism by commensal gut microbiota. These uremic toxins are considered to be risk factors for the progression and complications of CKD and impaired renal function [45]. In the present study, the metabolic profiles suggest a close relationship with gut microbial metabolism. Aromatic compounds such as benzenoids (Hydroxyphenylpyruvic acid, butylphenol), which are normally generated and biosynthesized by bacterial species, significantly differed between KT and control groups. Benzenoid compounds (Phenolic and indolic) are typical products of bacterial metabolism of aromatic amino acids, and dietary phenolic compounds are often transformed in the colon by the intestinal microbiota before absorption [46,47]. Clostridium and Eubacterium genera are considered as key players in this conversion [48]. The potential mechanistic participation of these metabolites requires further chemical elucidation. We believe that conducting further research to explore the potential role of benzenoids and other gut microbiota-derived metabolites in KT is warranted.

## 5. Conclusions

In this study, we have outlined a method that allows for an easy, non-invasive estimation of the recovery process of kidney-transplanted patients. We reported, for the first time, the advantage of fecal metabolomic assessment that could be a promising tool for revealing systematic metabolic variations related to renal graft. For instance, we performed a new method used for metabolome extraction that allowed us to cover a wide range of metabolite polarity. Moreover, the OPLS-DA analysis revealed high sensitivity to effectively distinguish between stable transplant recipients and healthy controls according to the fecal metabolic signature. Nevertheless, a logistic regression analysis was applied to assess the potential utility of altered metabolites as predictive markers. Interestingly, the most altered metabolites were long-chain fatty acids, phenolic compounds, and amino acids. Our preliminary results support the potential utility of fecal metabolome analysis in renal grafts that could improve current diagnostic methods and standards. The most important limitation of this methodology was that the use of different extraction solvents, devised to allow for large-scale quantitation of as many metabolites as possible, which might be time consuming compared to standard extraction procedures. The proposed protocol also needs a validation step for the characterized potential biomarkers. Finally, this large-scale multianalyte targeted approach was tested in fecal samples collected from a pilot kidney transplantation trial, with the aim of assessing the metabolomic coverage of this methodology in real samples and exploring its ability to investigate kidney transplantation metabolomic alterations as a case study and identify potential biomarkers. In this regard, future studies are needed to assess the clinical potential of this metabolomic platform with a larger sample of cohorts. Nevertheless, future studies are needed to enhance our understanding of the mechanisms underlying metabolomic abnormalities and gut microbial crosstalk in KT patients.

## Figures and Tables

**Table 1:** Clinical and demographic data of study groups.

Subjects	Age (Y) Mean ± SD	Gender	Diet	BMI ± SD	Immunosuppressive Therapy	Period (Y) after Tx Mean ± SD
Patients	42 ± 6	28 M/12 F	Low salt	23.7 ± 5	Str/Fk/MMF	6 ± 5
Controls	44 ± 5	10 M/10 F	Balanced	20 ± 4	-	-

(Y) year; SD Standard deviation; Str: steroids; FK: tacrolimus; MMF: mycophenolic acid; (-): not applicable; F: Female; M: Male; Tx: Treatment.

**Table 2:** Biomarkers selected in the fecal metabolic profiles of KT patients.

Extraction	RT	Metabolites	Sub Class	*m/z*	Chemical Structure	*p*-Value
**Ethanol**	2,4	Dodecanoic acid	Fatty acids	103.1/89.1/117.1	MF: C_12_H_24_O_2_ MW: 200.32 g/mol	<0.001
	5,5	Ethyl oleate	Dithianes	43.05/55.12/69.06	MF: C_20_H_38_O_2_ MW: 310.5 g/mol	<0.001
	8,9	trans-O-Dithiane-4,5-diol	Fatty acid ester	152.98/108.79/44.03	MF: C_4_H_8_O_2_S_2_ MW: 152.2 g/mol	<0.001
	9,8	Formic acid	Carboxylic acid	29.02/46.04/45	MF: CH_2_O_2_ MW: 46.025 g/mol	<0.001
	12,5	l-Tryptophan	Essential amino acid	130.07/159.09/232.06	MF: C_11_H_12_N_2_O_2_ MW: 204.22 g/mol	<0.001
	16,08	4-Hydroxyphenylpyruvic acid	Benzenoids	190.1/89.06/116.05	MF: C_9_H_8_O_4_ MW: 180.16 g/mol	<0.001
	16,2	5alpha-Cholest-7-en-3beta-ol	Sterols	43.05/386.33/255.22	MF: C_27_H_46_O MW: 386.7 g/mol	<0.001
	17,6	Valeric acid	Straight chain fatty acid	60.02/27.03/29.05	MF: C_5_H_10_O_2_ MW: 102.13 g/mol	<0.001
	18,02	Erucic acid	Long-chain fatty acid	321.2/303.3/255.1	MF: C_22_H_42_O_2_ MW: 338.6 g/mol	<0.001
**Ethyl Acetate**	4,1	Palmitic acid	Long-chain fatty acid	74/87/143	MF: C_16_H_32_O_2_ MW: 256.42 g/mol	<0.001
	10,5	Octadecanoic acid	Long-chain fatty acid	265.4/283.4/266.5	MF: C_18_H_36_O_2_ MW: 284.5 g/mol	<0.001
	13,48	Isostearic acid	Long-chain fatty acid	74.04/87.04/255.2	MF: C_18_H_36_O_2_ MW: 284.5 g/mol	<0.001
	13,9	Stigmastanol	Sterols	43.99/107.71/215.7	MF: C29H52O MW: 416.7 g/mol	<0.001
**Diethyl Ether**	3,8	5beta-Coprostanol	Cholestane steroids	81.09/95.02/67.1	MF: C_27_H_48_O MW: 388.7 g/mol	<0.001
	10,2	p-Anisic acid	Benzenoids	135.04/209.07/165.03	MF: C_8_H_8_O_3_ MW: 152.15 g/mol	<0.001
	11,5	Hentriacontane	Alkanes	71/85/99	MF: C_31_H_64_ MW: 436.8 g/mol	<0.001
	16,9	13-Methylmyristic acid	Long-chain fatty acid	73.05/89.04/43.05	MF: C_15_H_30_O_2_ MW: 242.4 g/mol	<0.001
**Chloroform**	4,03	2,4-Di-tert-butylphenol	Benzenoids	191.99/57.23/163.15	MF: C_14_H_22_O MW: 206.32g/mol	<0.001
	14,56	Androst-5-ene-3,17-dione	Androstane steroids	286/177/91	MF: C_19_H_26_O_2_ MW: 286.4 g/mol	<0.001
**Hexane**	8,8	Oleic Acid	Long-chain fatty acid	41/55/43	MF: C_18_H_34_O_2_ MW: 282.5 g/mol	<0.001
	12,6	Aspartylglycine ethyl ester	Alkyl-phenylketones	88.1/70.09/43.12	MF: C_8_H_14_N_2_O_5_ MW: 218.21 g/mol	<0.001

RT: retention time, MF: molecular formula, MW: molecular weight.

## Data Availability

Data available upon request from the first author, Soumaya Kouidhi (soumayakouidhi@gmail.com).

## Supplementary Materials

Supplementary materials are available online at https://www.mdpi.com/article/10.3390/diagnostics11060962/s1.
